# Retraction for: Multiwalled carbon nanotubes co-delivering sorafenib and epidermal growth factor receptor siRNA enhanced tumor-suppressing effect on liver cancer

**DOI:** 10.18632/aging.206013

**Published:** 2024-07-15

**Authors:** Zhili Wen, Yuliang Feng, Youwen Hu, Lingyan Lian, Hongyan Huang, Li Guo, Shanwen Chen, Qian Yang, Moran Zhang, Lijun Wan, Kedong Xu, Degejirifu, Xiaohua Yan

**Affiliations:** 1 Department of Gastroenterology, The Second Affiliated Hospital of Nanchang University, Nanchang, Jiangxi Province, China; 2 Jackson Laboratory for Genomic Medicine, Farmington, CT 06032, USA; 3 Department of Biochemistry and Molecular Biology, School of Basic Medical Sciences, Nanchang University, Nanchang, Jiangxi Province, China

**Keywords:** multiwalled carbon nanotubes, sorafenib, epidermal growth factor receptor, liver cancer

**This article has been retracted:** Aging has completed its investigation of this paper. Multiple concerns were raised about this paper, including internal duplications and overlap with unrelated papers from different institutions. We found that **Figure 1D**, “Gel retardation assay of naked siRNA and MWNT/Sor/siRNA complexes with the different N/P ratio,” is a duplication of the previously published Figure 2A, “Gel retardation assay of naked pDNA and SLNs/pDNA complexes with the different mass ratio” [1]. In addition, external duplications include images of immunohistochemical staining of Ki67 in **Figure 5D,** which duplicates Ki67 images in [2], and tumor images in **Figure 5C**, which are identical to some of the tumor images in [3, 4]. This figure also contains internal duplications produced by the repeated use of tumor specimens. Additionally, this figure has the same distinctively scratched ruler that was used to measure xenograft tumors in a number of other papers from unrelated teams of authors [5, 6]. Aging Scientific Integrity office contacted the authors but did not receive any response. It also notified authors’ Institutions about this retraction and added their names to the Editorial Warning list.
